# Coronary CT FFR vs Invasive Adenosine and Dobutamine FFR in a Right Anomalous Coronary Artery

**DOI:** 10.1016/j.jaccas.2022.06.009

**Published:** 2022-08-03

**Authors:** Marius R. Bigler, Anselm W. Stark, Andreas A. Giannopoulos, Adrian T. Huber, Matthias Siepe, Alexander Kadner, Lorenz Räber, Christoph Gräni

**Affiliations:** a Department of Cardiology, Inselspital, Bern University Hospital, University of Bern, Bern, Switzerland; b Cardiac Imaging, Department of Nuclear Medicine, University Hospital Zurich, Zurich, Switzerland; c Cardiology Department, University Hospital Zurich, Zurich, Switzerland; d Department of Radiology, Inselspital, University Hospital, University of Bern, Bern, Switzerland; e Department of Cardiovascular Surgery, Inselspital, Bern University Hospital, University of Bern, Switzerland

**Keywords:** anomalous coronary artery originating from the opposite sinus of Valsalva, computational fluid dynamics, coronary computed tomography angiography, fractional flow reserve, ACAOS, anomalous coronary artery originating from the opposite sinus of Valsalva, CCTA, coronary computed tomography angiography, CT, computed tomography, ECG, electrocardiogram, FFR, fractional flow reserve, HR, heart rate

## Abstract

We present the management of an anomalous coronary artery originating from the opposite sinus of Valsalva with comprehensive diagnostic workup including noninvasive coronary computed tomography (CT) derived fractional flow reserve (FFR) and invasive dobutamine-volume challenge-FFR/intravascular ultrasound. After surgical operation, treatment success was quantified by anatomical and functional analysis in postoperative CT. (**Level of Difficulty: Advanced.**)

## Central Illustration

**Figure:**
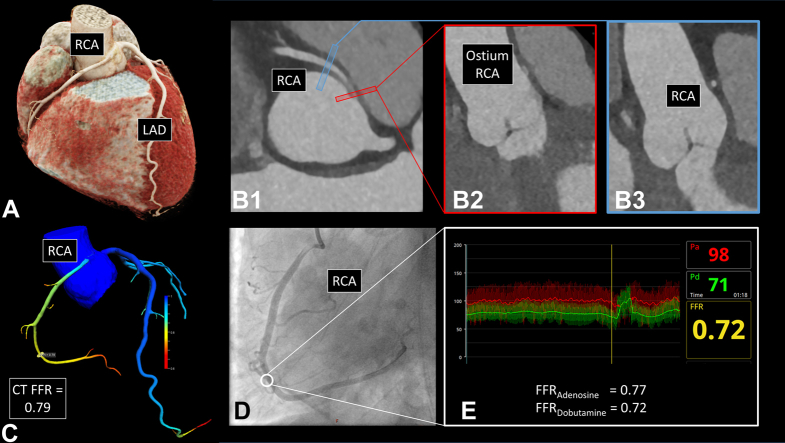

A 48-year-old man with known cardiovascular risk factors for dyslipidemia, smoking, a positive family history, and new onset of atypical chest pain for the previous 6 months during psychological stress was referred for further diagnostic workup at our tertiary center. In addition, intermittent dyspnea and palpitations were described. The electrocardiogram (ECG) was normal, and echocardiography showed normal left and right heart dimensions and function. The laboratory findings were unremarkable. The patient had performed competitive sports (ie, judo) in his youth and currently engaged in 9 hours of exercise per week without any previous exercise-related symptoms. A bicycle exercise test did not provoke any symptoms or ischemia-induced ECG changes at a maximal heart rate (HR) of 167 beats/min (corresponding to 97% of the predicted maximal HR by the formula 220 − age) and a maximal performance of 189 W (=105% of the maximum predicted).

**Learning Objectives**

• To understand the pathophysiology of ACAOS with fixed and dynamic stenotic components and the role of noninvasive and invasive diagnostic modalities in the comprehensive assessment of patients with ACAOS.
• To recognize the advantages, disadvantages, and diagnostic potential of fractional flow reserve calculations based on coronary computed tomography images in ACAOS for decision making towards downstream testing, therapy, and documentation of postsurgical success.

## Differential Diagnosis

**Figure 1:**
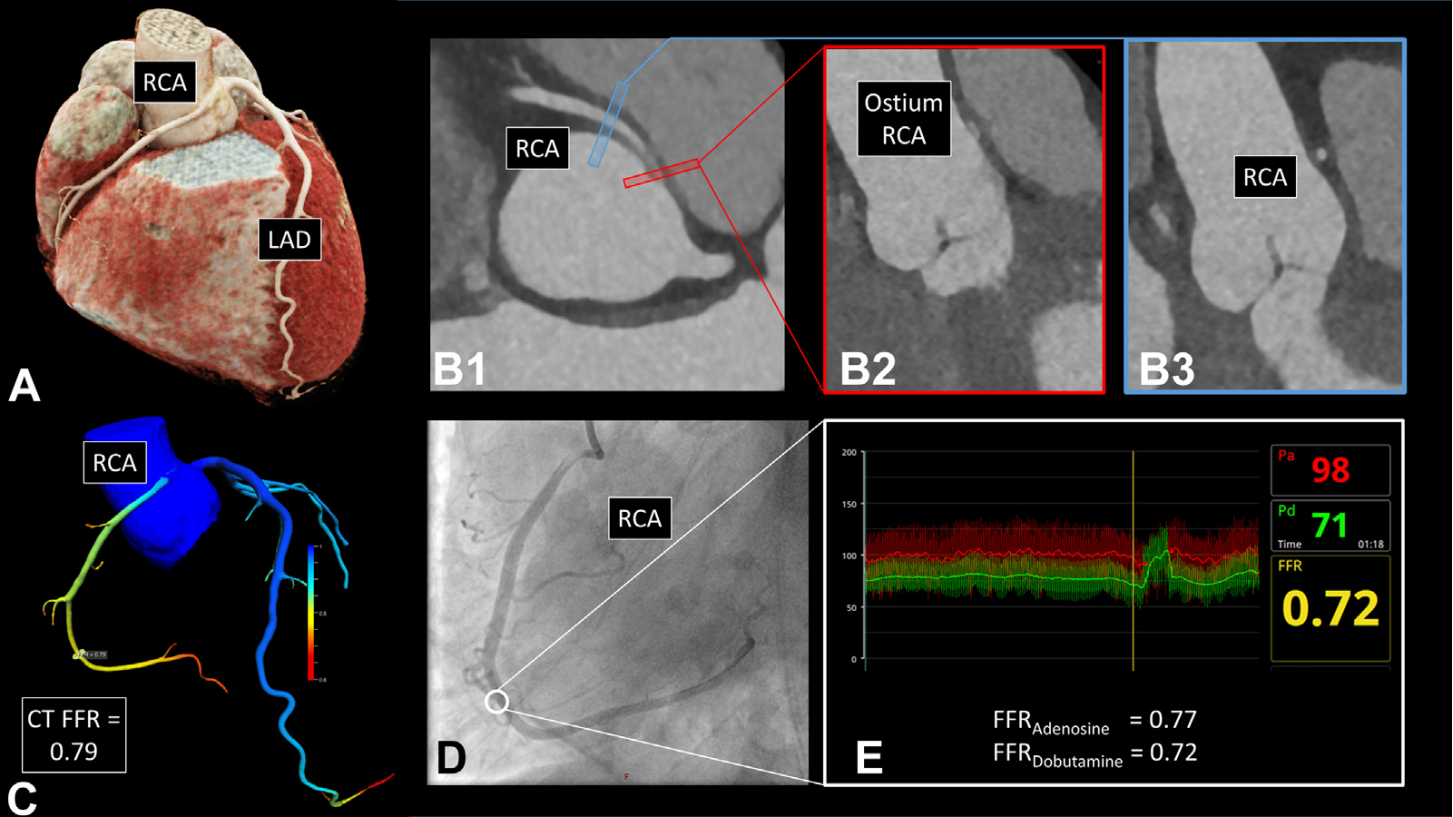
Preoperative Diagnostic Assessment **(A)** Three-dimensional reconstruction of coronary computed tomography angiography (CCTA) illustrating the anomalous origin of the right coronary artery (RCA). **(B1)** CCTA of the right anomalous coronary artery originating from the opposite sinus of Valsalva (R-ACAOS) illustrating the intramural course. **(B2)** Depiction of the R-ACAOS origin demonstrating the slitlike ostium at the beginning of the intramural course. **(B3)** Distal reference diameter of the RCA. **(C)** CCTA-derived fractional flow reserve (CT FFR). The CT FFR value right after the ostium was 0.89, further declining to 0.79 owing to the long intramural course with proximal narrowing and oval vessel shape. **(D)** Invasive coronary angiography with selective intubation of the R-ACAOS. **White circle** shows the location of the pressure sensor. **(E)** Invasive hemodynamic assessment, ie, FFR during dobutamine-volume challenge. LAD = left anterior descending (coronary artery); Pa = aortic pressure; Pd = distal pressure (measured by the pressure wire within the coronary artery).

To rule out coronary artery disease (CAD), coronary computed tomography angiography (CCTA) on Siemens Somatom Definition Flash was performed, which demonstrated no coronary plaques and no calcifications. However, a right anomalous coronary artery originating from the opposite sinus of Valsalva (right ACAOS), originating from the left coronary cusp with an interarterial course (ie, between the aorta and the pulmonary trunk), traditionally termed “malignant coronary artery anomaly” was detected with right coronary dominance (Figure 1). Beside the interarterial course, an intramural (ie, within the tunica media of the aortic root) course was found.

## Investigations

Quantitative assessment of the CCTA images showed anatomical high-risk features, including acute takeoff angle (3.9°, acute = <45°,^1^ slitlike ostium (56%), elliptic vessel shape of the proximal part with an elliptic ratio of 3.4^2^, and a long intramural course of 14.3 mm). There was no significant proximal stenosis (44% lumen narrowing). Furthermore, CT fractional flow reserve (FFR) (on-site software prototype cFFR, version 3.5.0 Siemens Healthineers) was performed and indicated a borderline hemodynamic relevance of the anomalous vessel (FFR = 0.79, threshold ≤0.80).

The patient subsequently underwent invasive coronary angiography with comprehensive invasive physiological evaluation, which confirmed the suspected hemodynamic relevance based on CT FFR measurements. In fact, invasive coronary angiography showed normal resting hemodynamic indices, but a relevant stenosis under adenosine infusion (140 μg/kg per body weight/min) with an FFR_Adenosine_ of 0.77. Furthermore, the dobutamine/volume challenge to simulate strenuous physical exercise (maximum dobutamine dose of 40 μg/kg per body weight/min, 3,000 mL ringer lactate, and 1 mg atropine)^3^ additionally accentuated the hemodynamic significance, resulting in an FFR_Dobutamine_ of 0.72 (maximal HR = 154/min = 90%). Simultaneously performed intravascular ultrasonography confirmed the significance of the anomaly with a lateral dynamic compression of the vessel (reduction of the minimal lumen area of 5.34 to 3.26 mm^2^, ie, 39% under maximal dobutamine/volume stress (Supplemental Figure 1).

## Management

**Figure 2:**
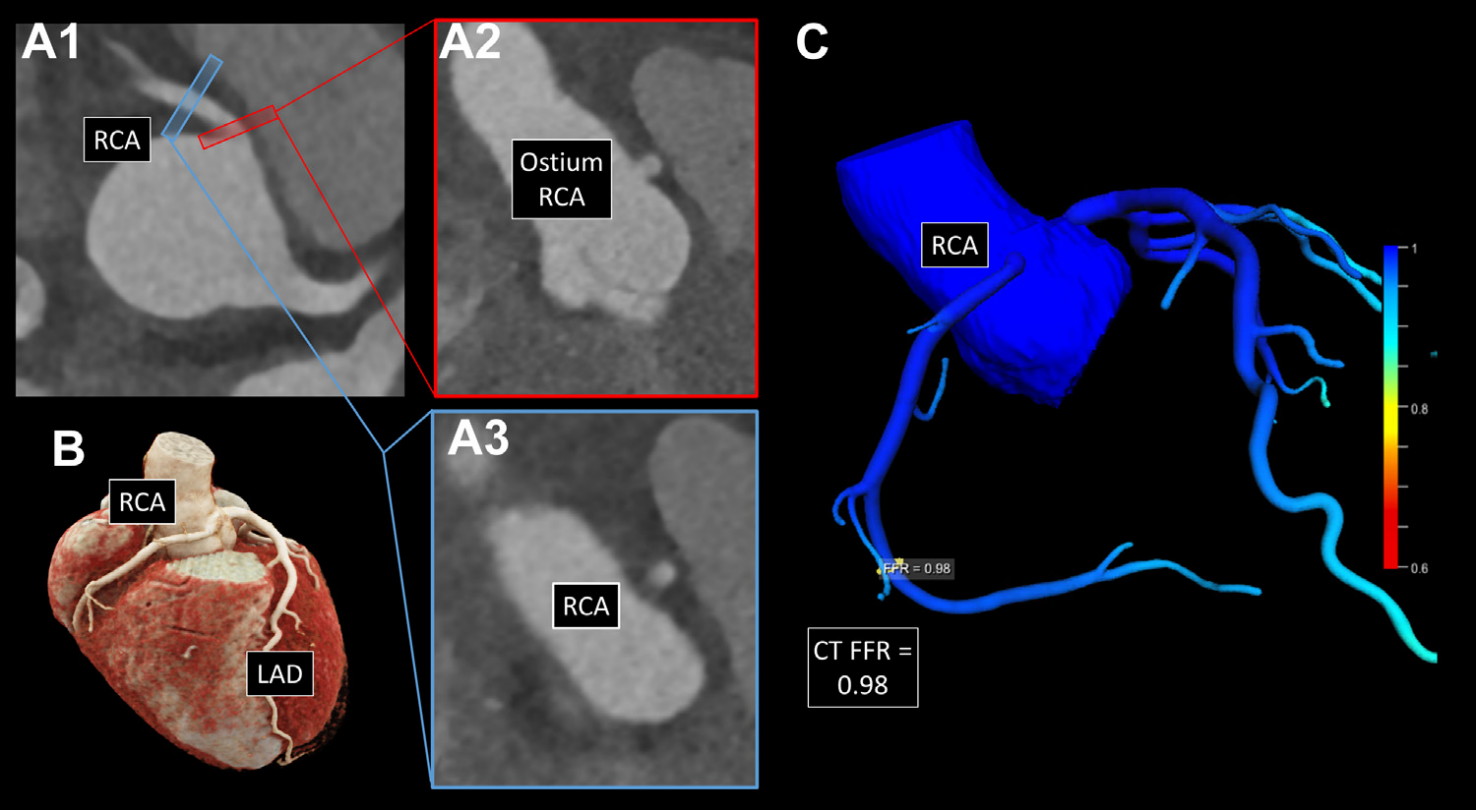
Postoperative Diagnostic Evaluation **(A1)** Coronary computed tomography angiography (CCTA) of the right anomalous coronary artery originating from the opposite sinus of Valsalva (R-ACAOS) after surgical unroofing. **(A2)** Depiction of the R-ACAOS origin demonstrating successful correction of the slitlike ostium. **(A3)** Unchanged distal reference diameter of the right coronary artery (RCA). **(B)** Three-dimensional reconstruction of the CCTA without visible external alterations because of the applied surgical procedure, ie, surgical unroofing of the intermural course. **(C)** Postoperative CCTA-derived fractional flow reserve (CT FFR) demonstrating the hemodynamic success of the surgical procedure.

After interdisciplinary discussion by the heart team, surgical treatment with unroofing of the intramural course was successfully performed. Three days after the operation, repeated CCTA revealed a successful improvement of all anatomical high-risk features (takeoff angle 24.6°, slitlike ostium 15.4%, proximal stenosis 30.8%, elliptic vessel shape ratio of 1.25, and intramural course length 0.0 mm), resulting in a normalized CT FFR of 0.98 (Figure 2).

## Follow-Up

The patient underwent 12 weeks of outpatient cardiac rehabilitation, presenting free of any symptoms at the 4-month follow-up visit. In addition, a follow-up bicycle exercise test demonstrated increased clinical efficacy without any symptoms or ischemia-induced ECG changes at a maximal HR of 163 beats/min (= 95% of maximal HR) and a maximal performance of 213 W (=118%, +13% compared with initial test result).

## Discussion

We present the case of a middle-aged man with atypical cardiac symptoms and right ACAOS with anatomical high-risk features of unclear clinical significance. Although direct treatment would be justifiable according to the current guidelines, which recommend surgical revascularization with a low threshold,^4^ recent data have shown a favorable midterm outcome in a middle-aged population with newly diagnosed ACAOS.^5^ Therefore, the evaluation of anatomical high-risk features as well as the coronary dominance is an important step. Furthermore, evaluation of the hemodynamic relevance to prevent unnecessary open-heart surgery of a benign coincidental ACAOS finding is the crucial step in the decision making for the management of such anomalies. An important question in this clinical setting is the following: Is it generally possible that a person with lifelong ACAOS and without any symptoms during youth, even during competitive sports, can present with a first onset of ACAOS-related symptoms later in life?^6^ It is indeed plausible that age-dependent alteration of the aorta (ie, dilatation, stiffening of the aortic wall) could slowly aggravate the anatomical high-risk features of ACAOS. In such situations with unclear symptoms, a comprehensive noninvasive and invasive assessment is even more important to avoid unnecessary interventions. As in our case, CT FFR and adenosine FFR showed borderline hemodynamic relevance of the ACAOS, which was aggravated by invasive dobutamine-volume challenge, and the interdisciplinary decision was made for surgical correction, which led to a complete symptom-free patient with increased maximal physical performance postoperatively and documented success in the CCTA and CT FFR.

The complex pathomechanisms of myocardial ischemia in ACAOS with a fixed and a dynamic component require a comprehensive hemodynamic evaluation beyond the assessment of the anatomical high-risk features. Whereas the fixed component (represented by such anatomical high-risk features as slitlike ostium and proximal narrowing) has similarities to atherosclerotic lesions, the dynamic stenotic component (ie, acute takeoff angle and intramural course with lateral compression) occurs only during strenuous physical exercise promoted by aortic dilation and reduced diastolic perfusion time. Additionally, the mass of viable myocardium, the distensibility of the aortic wall, and the volume status of the patient directly affects the hemodynamic relevance.

Therefore, the evaluation of the fixed component is similar to that for atherosclerotic lesions and can be performed by the use of vasodilator pharmacological stress (ie, adenosine), whereas the dynamic component has to be assessed by supramaximal stress testing (eg, physical stress or dobutamine), including elevation of the systolic pressure, stroke volume, and heart rate. One has to be aware that physical stress would be the preferred modality, and dobutamine stress represents only a simulation and approximation to physical stress with certain limitations (eg, preload reduction, which has to be counterbalanced with volume). In our case, a relevant fixed component was found, which was represented by the pathological invasive FFR_Adenosine_ and CT FFR. CT FFR is validated against FFR_Adenosine_ in atherosclerotic lesions^7^ and allows a simulation of the pressure gradient during hyperemia from a coronary CT scan under resting conditions. Consequently, CT FFR seems to be able to assess only the fixed stenotic components, similar to invasive FFR_Adenosine_. Inasmuch as under maximal dobutamine volume challenge (ie, FFR_Dobutamine_), the hemodynamic relevance increased, it seems that besides the relevant fixed component, the additional dynamic components played a role in the hemodynamic relevance in this particular case. In fact, FFR_Dobutamine_ might be the ideal protocol to assess both the fixed and the dynamic components, inasmuch as it captures not only the dynamic stenotic component but also the fixed stenosis because of an increased myocardial oxygen demand with associated physiological vasodilatation. Hence, FFR_Dobutamine_ is usually more severe or equal to FFR_Adenosine_ depending on the extent of the dynamic stenotic component.^3^ However, one could argue that in an ACAOS case with pathological CT FFR, a further invasive measurement with extensive dobutamine volume protocol is not necessary because the hemodynamic relevance of the fixed component of the ACAOS is already proven. Therefore, it can be discussed whether, depending on the findings, CT FFR may act indeed as a gatekeeper toward the decision of additional downstream testing. Furthermore, after successful correction of all dynamic components during cardiac surgery, CT FFR could possibly help in the evaluation of postsurgical success. However, until the application of CT FFR in patients with ACAOS becomes routine, profound clinical data must be collected in this specific condition.

## Conclusions

This case illustrates the contemporary management of an anomalous coronary artery originating from the opposite sinus of Valsalva, which includes coronary computed tomography angiography and invasive dobutamine volume challenge. Further, CT FFR calculations from noninvasive imaging may help toward optimal downstream testing, therapy decision, and documentation of postsurgical success. However, given that current noninvasive CT FFR measurement is limited to the evaluation of the fixed component, novel simulation models should include additional dynamic components of ACAOS with validation against invasive FFR_Dobutamine_.

## Funding Support and Author Disclosures

Dr. Gräni has received funding from the Swiss National Science Foundation Grant Number 200871, the Swiss National Foundation, InnoSuisse, and the Center of Artificial Intelligence in Medicine Bern. Dr. Huber has received research grants from the Swiss National Science Foundation, the Swiss Academy of Medical Sciences, the Helmut-Hartweg Foundation, and the Foundation to Fight against Cancer, all for work outside the submitted study; and he has received speaker/consulting honoraria or travel support from Bayer, Bracco, and Siemens, all for work outside the submitted study. All other authors have reported that they have no relationships relevant to the contents of this paper to disclose.
