# Supplementary material for: Coronary CT FFR vs Invasive Adenosine and Dobutamine FFR in a Right Anomalous Coronary Artery
Source: JACC Case Rep. 2022 Aug 3;4(15):929–33. doi: 10.1016/j.jaccas.2022.06.009 (PMC9350890; doi:10.1016/j.jaccas.2022.06.009)

**Supplemental Figure 1 Intravascular Ultrasound Image of the Intramural Course**

Smallest cross-sectional area **(A)** at rest (5.34 mm^2^) and **(B)** during maximal heart rate at the end of the dobutamine-volume challenge (3.26 mm^2^). The initial oval vessel shape is further aggravated during maximal stress (ie, lateral compression). **White dotted line** = outline of the coronary artery.


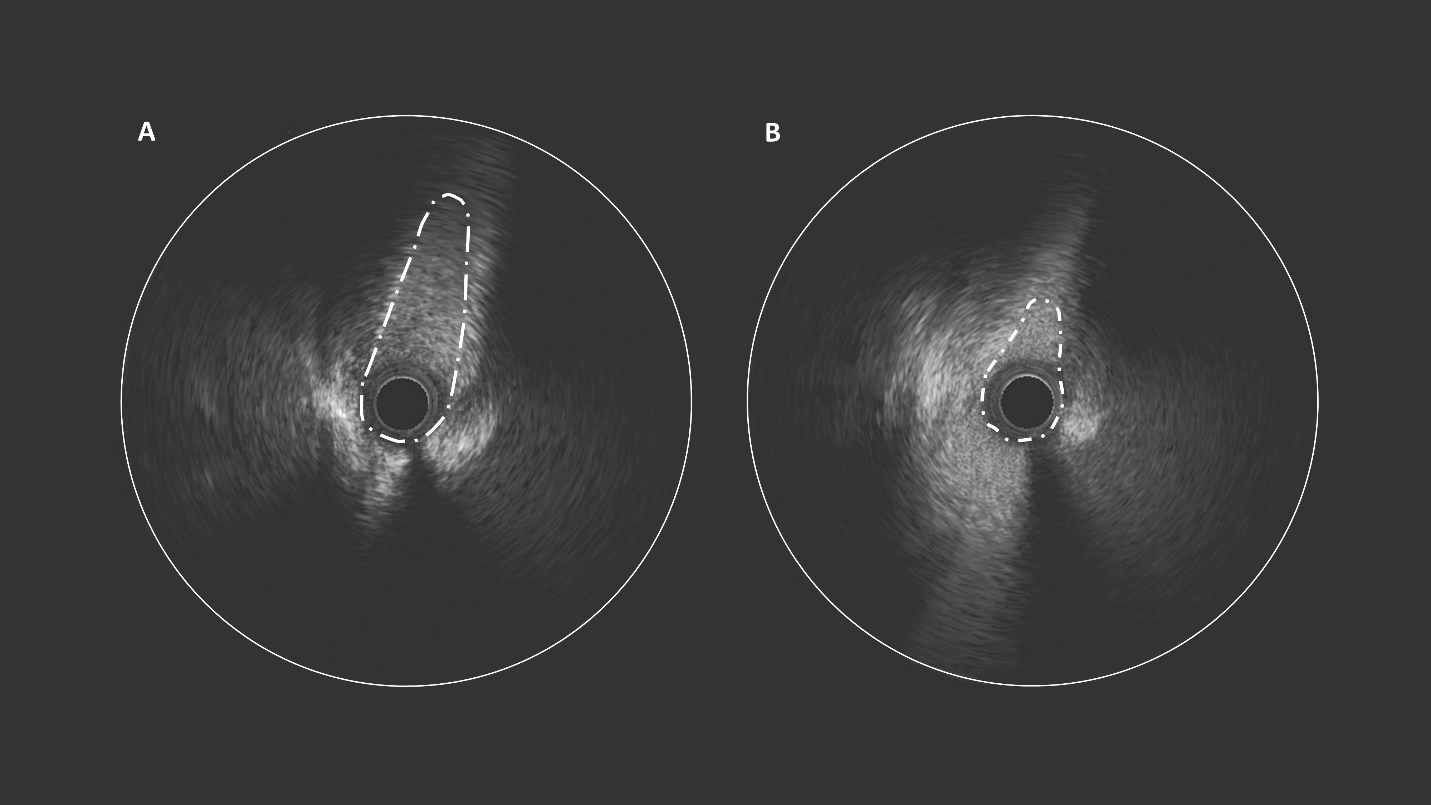

Supplement: Supplemental Figure 1 [file mmc1.docx]
